# *Wolbachia w*Mel strain-mediated effects on dengue virus vertical transmission from *Aedes aegypti* to their offspring

**DOI:** 10.1186/s13071-023-05921-y

**Published:** 2023-08-31

**Authors:** Kien Duong Thi Hue, Daniela da Silva Goncalves, Vi Tran Thuy, Long Thi Vo, Dui Le Thi, Nhu Vu Tuyet, Giang Nguyen Thi, Trang Huynh Thi Xuan, Nguyet Nguyen Minh, Phong Nguyen Thanh, Sophie Yacoub, Cameron P. Simmons

**Affiliations:** 1 Oxford University Clinical Research Unit, Wellcome Trust Africa Asia Programme, District 5, Ho Chi Minh City, Vietnam; 2 Hospital for Tropical Diseases, District 5, Ho Chi Minh City, Vietnam; 3 Centre for Tropical Medicine and Global Health, University of Oxford, Oxford, UK; 4 Institute for Vector Borne Disease, Monash University, Clayton Campus, Melbourne, VIC 3168 Australia

**Keywords:** Vertical transmission, Dengue virus, Mosquitoes, *Aedes aegypti*, *Wolbachia*, *w*Mel

## Abstract

**Background:**

Dengue virus serotypes (DENV-1 to -4) can be transmitted vertically in *Aedes aegpti* mosquitoes. Whether infection with the* w*Mel strain of the endosymbiont *Wolbachia* can reduce the incidence of vertical transmission of DENV from infected females to their offspring is not well understood.

**Methods:**

A laboratory colony of Vietnamese *Ae. aegypti*, both with and without *w*Mel infection, were infected with DENV-1 by intrathoracic injection (IT) to estimate the rate of vertical transmission (VT) of the virus. VT in the DENV-infected mosquitoes was calculated via the infection rate estimation from mosquito pool data using maximum likelihood estimation (MLE).

**Results:**

In 6047 F1 Vietnamese wild-type *Ae. aegypti*, the MLE of DENV-1 infection was 1.49 per 1000 mosquitoes (95% confidence interval [CI] 0.73–2.74). In 5500 *w*Mel-infected *Ae. aegypti*, the MLE infection rate was 0 (95% CI 0–0.69). The VT rates between mosquito lines showed a statistically significant difference.

**Conclusions:**

The results reinforce the view that VT is a rare event in wild-type mosquitoes and that infection with *w*Mel is effective in reducing VT.

**Graphical Abstract:**

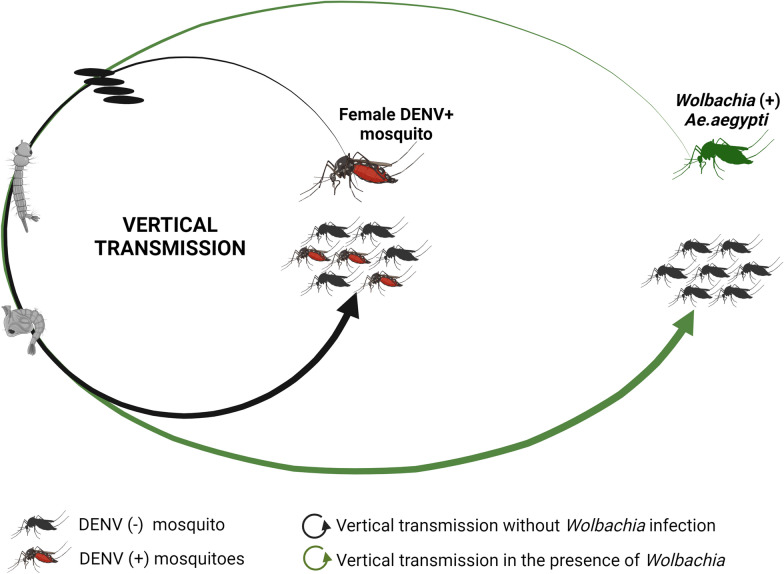

**Supplementary Information:**

The online version contains supplementary material available at 10.1186/s13071-023-05921-y.

## Background

Dengue is a mosquito-borne viral infection caused by one of four dengue virus serotypes (DENV-1 to -4) that is endemic in many tropical and sub-tropical countries [1]. The global incidence of dengue has increased dramatically in the last 50 years, with approximately 50–100 million symptomatic infections and 20,000 deaths reported annually in over 125 countries [2, 3]. DENV is transmitted between humans through the bite of an infected female *Aedes* sp. mosquito (*Ae. aegypti* or *Ae. albopictus*). However, DENV can also be transmitted vertically, from the DENV-infected female mosquito to her offspring during follicle development or oviposition [4–6]. This latter transmission mode is hypothesized to contribute to DENV persistence in the mosquito population [7]. A sign of vertical transmission (VT) of DENV in a mosquito populations is the presence of infected male mosquitoes (which do not blood feed) and the presence of viruses in immature forms of mosquitoes of any sex. VT is a rare event in nature [8–11] but it has been observed in laboratory studies in both *Ae. aegypti* and *Ae. albopictus* [12, 13]. Several studies have also shown that VT is influenced by a number of factors, including mosquito-rearing temperature, mosquito strain and virus strain [14–18].

Although there are two licensed dengue vaccines, vector control has been the mainstay of dengue control efforts for decades. However, it is obvious that existing vector control methods have not removed the public health burden of dengue in any endemic country. A new approach that involves the insect endosymbiont *Wolbachia* (*w*Mel or *w*AlbB strains) is being applied to render *Ae. aegypti* populations much less competent at transmitting DENV between humans [19–21]. *Wolbachia* inhibits virus replication in mosquito cells via multiple mechanisms, including altering the intracellular environment, activating the innate immune system and interacting with the cell machinery involved in RNA virus infection [22]. Multiple epidemiological studies, including a cluster randomized trial, have shown a large decrease in dengue cases in communities with* w*Mel-*Wolbachia*-treated mosquitoes, demonstrating that *w*Mel introgression is an effective disease control measure [23, 24].

We previously found a very low DENV VT rate (0.23%) among wild-type (Wt) *Ae. aegypti* orally infected with DENV after feeding on viremic blood from dengue patients [9]. To assess whether *w*Mel would eliminate VT of DENV, we established an experimental model system to measure VT in the presence and absence of *w*Mel infection.

## Methods

### Mosquito lines and rearing

The *w*Mel-infected *Ae. aegypti* population (*w*Mel-*Ae. aegypti*; Vietnamese genetic background) was generated by backcrossing, as previously described [25]. Generations G59 to G61 of colonized *w*Mel-*Ae. aegypti* and generations F54 and F55 of colonized Wt *Ae. aegypti* were used in this study, as previously described [9, 26]. The presence of *w*Mel in each *w*Mel-*Ae. agypti* generation was confirmed by testing [27]. The mosquitoes were reared and maintained under laboratory conditions, at 26–28 °C, 65–85% relative humidity, and a 12:12-h light/dark cycle, with access to 10% sucrose solution ad libitum.

### Generating DENV-infected F0 mosquitoes

Both Wt and *w*Met-infected adult female mosquitoes, aged 2–3 days, were injected intrathoracically with 1 µl of solution containing DENV-1 grown in cell culture (10^5^ pfu/ml; Genbank Accession Number: FJ432735). DENV-1 was used to establish infection based on the findings of our previous study which indicated that this serotype was less inhibited by *w*Mel than the other three DENV serotypes [28]. Ten injected females were then kept in cups containing 10 male mosquitoes (female:male ratio = 1:1) where they were maintained on sucrose for 10 days.

A human blood meal (non-infectious blood provided via a membrane feeder) was provided to surviving F0 females on day 10 post-injection [28]. After 30 min of blood feeding, fully engorged females were isolated and placed into separate cups (isofemales) containing wet cotton balls for oviposition. Sugar (a piece of cotton soaked in 10% sucrose) was provided for 14 days. On day 14 post non-infectious blood meal, individual F0 females were harvested for testing of their DENV infection status.

### Hatching and harvesting F1 mosquitoes

F1 eggs were collected at 5–7 days after the F0 females had taken their non-infectious blood meal and placed in trays filled with fresh water; the trays were kept in incubators at 28 °C under a 12/12-h light/dark cycle. Each tray was provided with one 100 mg tablet of fish food. The larval density was maintained at approximately 3300–3500 larvae per 1.5 l of water. F1 mosquitoes were individually stored and sorted by sex within cohorts originating from the same mother.

F0 and F1 mosquitoes homogenized in a TissueLyser II instrument (Qiagen, Hilden, Germany) at 30 Hz for 2–5 min. Each F0 mosquito homogenate was stored individually, while F1 mosquito homogenates (50 µl from each sample) were pooled before conducting the reverse transcriptase PCR (RT-PCR) assays to detect the presence of *Wolbachia* [27] and DENV [29]. The positive pooled samples were then un-pooled to determine the number of infected individuals using the remaining volume of mosquito homogenate.

### Estimating VT of DENV-1 in colonized *Ae. aegypti* with and without *w*Mel infection

To estimate the VT of DENV-1 in a large number of F1 mosquitoes, we utilized the maximum likelihood estimate (MLE) method. This method estimated the proportion of DENV-infected individuals in pooled samples, defined as the infection rate most likely observed given the test results and an assumed probabilistic model (binomial distribution of infected individuals in a positive pool) [30–35]. To account for biases, we utilized bias-corrected likelihood methods and calculated a skew-corrected score confidence interval (95% CI) [36]. The infection rate was reported as the number of infected mosquitoes per 1000 individuals. In addition, to perform comparisons of CIs, we utilized the Wilson score-based interval of the Newcombe method, which relies on exact calculations of coverage probabilities [37]. This approach allowed us to assess the statistical significance of the differences in infection rates between populations.

To accurately estimate the proportion of infected individuals in a population using MLE, determining the appropriate pool size is crucial to minimize the likelihood of false-negative results. In this study, the pool size was examined using a sample-media pooling approach, adapted from the Clinical and Laboratory Standards Institute (CLSI) document EP12 (CLSI EP12 [38]). The positive percent agreement (PPA) and negative percent agreement (NPA) values were calculated for different pool sizes. The highest PPA value (96%) was obtained with a pool size of four mosquitoes, whereas a pool size of eight mosquitoes showed a PPA of 89% (95% CI 0.8–0.9) and a pool size of sixteen showed only 68% agreement [39]. All three sizes showed 100% agreement fir NPA values. Therefore, a pool size of eight was selected [39].

## Results and discussion

**Fig. 1:**
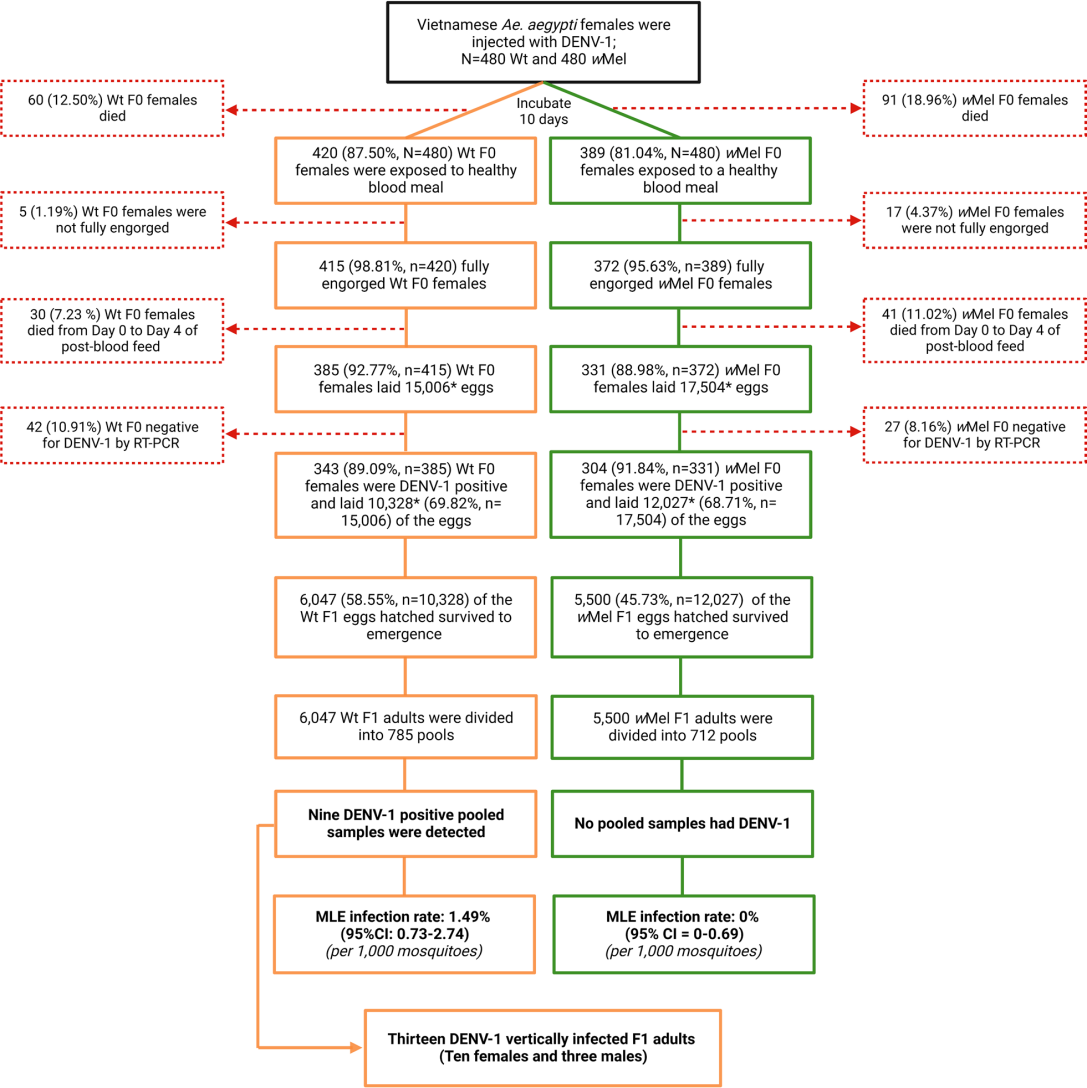
Flowchart of vertical transmission estimation of DENV-1 in Wt and *w*Mel-infected *Ae. aegypti* with a Vietnamese background. Shown is the fate of mosquitoes as they were processed in order to determine the frequency of vertical transmission of F0 females after being infected by microinjection of DENV-1 (strain FJ432735). The orange boxes represent Wt *Ae. aegypti*, the green boxes represent *w*Mel-*Ae. aegypti* and the red boxes indicate excluded samples. The asterisk (*) indicates the numbers are estimated. DENV-1, Dengue virus serotype 1; MLE, maximum likelihood estimate of infection rate; RT-PCR, reverse transcriptase-PCR; *w*Mel, *Wolbachia* strain *w*Mel-infected *Ae. aegypti*; Wt, wild type

**Fig. 2:**
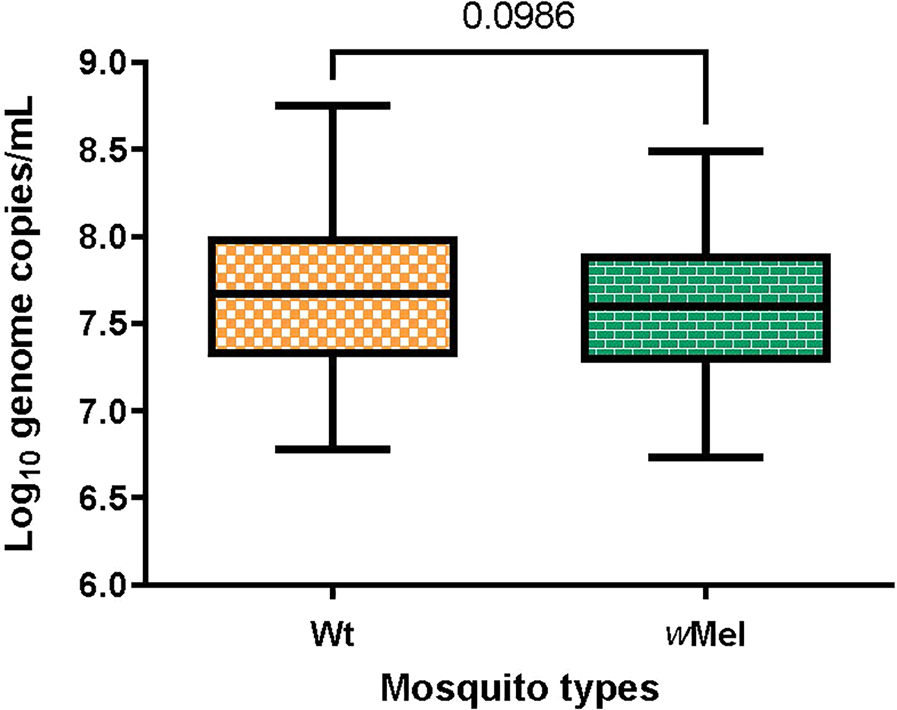
DENV-1 copy numbers in F0 female *Ae. aegypti* with and without *w*Mel infection. The infectivity of DENV-1 by intrathoracic injection in F0 females represents log10 viral genome copy numbers. Wt mosquitoes are shown in orange and *w*Mel-mosquitoes are shown in green. *w*Mel, *Wolbachia* strain *w*Mel-infected *Ae. aegypti*; Wt, wild type

Our study aimed to measure whether VT was less likely to occur in *w*Mel-infected *Ae. aegypti* mosquitoes (*w*Mel-*Ae. aegypti*) versus their Wt counterparts. The estimation of VT was conducted in 480 *w*Mel-*Ae. aegypti* females and in 480 Wt females (Fig. 1). Ten days after intrathoracic (IT) inoculation of DENV-1, 420 (87.50%, *N* = 480) Wt and 389 (81.04%, *N* = 480) *w*Mel-*Ae. aegypti* mosquitoes survived and were given a non-infectious blood meal. Between 5 and 7 days later, approximately 10,328 eggs were collected from 343 (89.09%, *n* = 385) virus-infected Wt F0 females (out of 385 Wt F0 females that survived and laid eggs), and approximately 12,027 F1 eggs were collected from 304 (91.84%, *n* = 331) virus-infected *w*Mel-*Ae. aegypti* F0 females (out of 331 *w*Mel-*Ae. aegypti* F0 females that survived and laid eggs). In both mosquito lines, high parental infection rates of DENV-1 were observed, with 89.09% (343/385, surviving mosquitoes only) of Wt mosquitoes infected and 91.84% (304/331, surviving mosquitoes only) of *w*Mel-*Ae. aegypti* mosquitoes infected, respectively (Fig. 1). Consistent with previous findings, IT injection resulted in a higher prevalence of DENV-1 compared to oral feeding [9, 40]. DENV-1 RNA concentrations in whole bodies of F0 Wt and *w*Mel-*Ae. aegypti* were comparable and high: 7.7 (95% CI 7.63–7.72) and 7.6 (95% CI 7.57–7.67) log_10_ copies/ml, respectively (Fig. 2). The DENV copy numbers detected in the whole bodies of each mosquito line were not significantly different (Mann–Whitney test: *U* = 48216, *P* = 0.09, 95% CI = -0.01-0.13), possibly due to the IT inoculation with a large inoculum of the virus.

A total of 6047 F1 adults emerged from the approximately 10,328 eggs collected from 343 virus-infected Wt F0 females. Similarly, the approximately 12,027 F1 eggs from 304 virus-infected *w*Mel-*Ae. aegypti* F0 females completed their development, providing 5500  *w*Mel-*Ae. aegypti* F1 adults (Fig. 1). All Wt F1 adult mosquitoes were grouped into 785 pools, and 5500 *w*Mel-*Ae. aegypti* F1 adult mosquitoes were grouped into 712 pools (for details, see Additional file 1: Table S1). These pools were tested for DENV-1, and nine positive pools were detected from the Wt group, while no positive pools were found in the *w*Mel-*Ae. aegypti* group. The MLE for the VT rate of DENV-1 in Wt mosquitoes was estimated to be 1.49 (95% CI 0.73–2.74) per 1000 adults. However, in *w*Mel-*Ae. aegypti* mosquitoes, the transmission rate was estimated to be zero (95% CI 0–0.69). The observed difference in the infection rates between the Wt group and the *w*Mel-*Ae. aegypti* group is 1.49 (95% CI 0.67–5.05). As the 95% CI is entirely above 0, the null hypothesis of no difference (H0: Wt − *w*Mel = 0) can be rejected at a significance level of *P* < 0.05. Consequently, it can be concluded that the VT rate of the Wt group is significantly higher than that of the *w*Mel group.

Thirteen DENV-1 infected Wt F1 individuals were identified from the nine positive pools, of which 10 were females. When assessing the frequency of mothers transmitting the virus to their progeny and determining the proportion of progeny born to DENV-infected mothers [9] based on positive pools, we observed a slight increase in rates compared to those found in field-collected mosquitoes [9]. Our estimates indicated that for every 343 DENV-infected Wt *Ae. aegypti* female mosquitoes that survived and laid eggs, 11 individuals (3.21%) transmitted the virus to their offspring. However, experimentally we found that only 0.13% of progeny born to DENV-infected mothers would be infected with DENV-1(13 out of 10,328 eggs acquiring the infection). This proportion increased to 0.21% when calculated based on 6047 F1 adults. The frequency of mothers transmitting the virus to any of their progeny in Wt *Ae. aegypti* (3.21%) compared to the frequency identified in field-collected *Ae. aegypti* (2.43%) using patient-derived blood meals [9] might be attributed to the direct introduction of virus into systemic tissues via IT inoculation.

The MLE of DENV-1 infection rate observed in *w*Mel-infected *Ae. aegypti* was comparable to that found in *w*Mel-infected *Ae. aegypti* from Brazil [40]. In our study, we optimized the conditions to increase the probability of a VT event in Wt *Ae. aegypti*, by employing IT inoculation of virus and a long extrinsic incubation period. Furthermore, our study was conducted with a large sample size, approximately 11,547 total F1 *Ae. aegypti* (6047 Wt and 5500 *w*Mel-*Ae. aegypti*). Despite these advantages, the VT event was not recorded in *w*Mel-infected *Ae. aegypti*. The absence of DENV infection in *w*Mel-infected F1 mosquitoes and the observed difference in VT rates between mosquito lines provide evidence that *w*Mel is effective in reducing VT. The ability of *w*Mel to decrease DENV replication in *w*Mel-carrying mosquitoes has been used to reduce the incidence of dengue through the deployment of *Wolbachia* mosquitoes in endemic areas [23, 24]. The ability of *w*Mel to reduce the VT of DENV can be attributed to various mechanisms, including resource competition, immune system activation and interference with viral replication in the reproductive tissues of mosquitoes [41–44]. These mechanisms are recognized for their ability to reduce the replication of DENV and could therefore limit its transmission from one generation to the next generation. The capacity of *w*Mel to diminish dengue transmission in both horizontal and vertical modes of transmission is critical in mitigating the incidence of dengue, ultimately decreasing the overall disease burden.

## Conclusions

The results of the present study support the belief that VT is a rare phenomenon. *w*Mel infection reduces VT in *w*Mel-carrying *Ae. aegypti* population.

### Supplementary Information

**Additional file 1: Table S1.** Number of F1 adults distributed in each pooled sample.

## Data Availability

All relevant data generated or analyzed during this study are included in this published article and its additional files.

## Abbreviations

DENV: Dengue virus
IT: Intrathoracic
MLE: Maximum likelihood estimate (of infection rate)
NPA: Negative percent agreement
RT-PCR: Reverse transcriptase PCR
PPA: Positive percent agreement
VT: Vertical transmission
*w*Mel: *Wolbachia pipientis*, *w*Mel strain
Wt: Wild type

## References

[1] Kraemer MU, Sinka ME, Duda KA, Mylne AQ, Shearer FM, Barker CM (2015). The global distribution of the arbovirus vectors *Aedes aegypti* and *Ae. albopictus*. Elife.

[2] Stanaway JD, Shepard DS, Undurraga EA, Halasa YA, Coffeng LE, Brady OJ (2016). The global burden of dengue: an analysis from the Global Burden of Disease Study 2013. Lancet Infect Dis.

[3] Messina JP, Brady OJ, Pigott DM, Brownstein JS, Hoen AG, Hay SI (2014). A global compendium of human dengue virus occurrence. Sci Data.

[4] Adams B, Boots M (2010). How important is vertical transmission in mosquitoes for the persistence of dengue? Insights from a mathematical model. Epidemics.

[5] Haddow AD, Guzman H, Popov VL, Wood TG, Widen SG, Haddow AD (2013). First isolation of *Aedes* flavivirus in the *Western* hemisphere and evidence of vertical transmission in the mosquito *Aedes* (Stegomyia) *albopictus* (Diptera: Culicidae). Virology.

[6] Arunachalam N, Tewari SC, Thenmozhi V, Rajendran R, Paramasivan R, Manavalan R (2008). Natural vertical transmission of dengue viruses by *Aedes aegypti* in Chennai, Tamil Nadu India. Indian J Med Res.

[7] Khin MM, Than KA (1983). Transovarial transmission of dengue 2 virus by *Aedes aegypti* in nature. Am J Trop Med Hyg.

[8] Grunnill M, Boots M (2016). How important is vertical transmission of dengue viruses by mosquitoes (Diptera: Culicidae)?. J Med Entomol.

[9] Goncalves DDS, Hue KDT, Thuy VT, Tuyet NV, Thi GN, Thi Thuy VH (2020). Assessing the vertical transmission potential of dengue virus in field-reared *Aedes aegypti* using patient-derived blood meals in Ho Chi Minh City, Vietnam. Parasit Vectors.

[10] Thangamani S, Huang J, Hart CE, Guzman H, Tesh RB (2016). Vertical transmission of Zika virus in *Aedes aegypti* mosquitoes. Am J Trop Med Hyg.

[11] da Costa CF, da Silva AV, do Nascimento VA, de Souza VC, Monteiro D, Terrazas WCM (2018). Evidence of vertical transmission of Zika virus in field-collected eggs of *Aedes aegypti* in the Brazilian Amazon. PLoS Negl Trop Dis.

[12] Joshi V, Mourya DT, Sharma RC (2002). Persistence of dengue-3 virus through transovarial transmission passage in successive generations of *Aedes aegypti* mosquitoes. Am J Trop Med Hyg.

[13] Gunther J, Martinez-Munoz JP, Perez-Ishiwara DG, Salas-Benito J (2007). Evidence of vertical transmission of dengue virus in two endemic localities in the state of Oaxaca, Mexico. Intervirology.

[14] Farnesi LC, Martins AJ, Valle D, Rezende GL (2009). Embryonic development of *Aedes aegypti* (Diptera: Culicidae): influence of different constant temperatures. Mem Inst Oswaldo Cruz.

[15] Hardy JL, Rosen L, Kramer LD, Presser SB, Shroyer DA, Turell MJ (1980). Effect of rearing temperature on transovarial transmission of St. Louis encephalitis virus in mosquitoes. Am J Trop Med Hyg.

[16] Mitchell CJ, Miller BR (1990). Vertical transmission of dengue viruses by strains of *Aedes albopictus* recently introduced into Brazil. J Am Mosq Control Assoc.

[17] Kramer LD, Ebel GD (2003). Dynamics of flavivirus infection in mosquitoes. Adv Virus Res.

[18] Buckner EA, Alto BW, Lounibos LP (2016). Larval temperature-food effects on adult mosquito infection and vertical transmission of dengue-1 virus. J Med Entomol.

[19] Hugo LE, Rasic G, Maynard AJ, Ambrose L, Liddington C, Thomas CJE (2022). *Wolbachia* wAlbB inhibit dengue and Zika infection in the mosquito *Aedes aegypti* with an Australian background. PLoS Negl Trop Dis.

[20] Ahmad NA, Mancini MV, Ant TH, Martinez J, Kamarul GMR, Nazni WA (1818). *Wolbachia* strain wAlbB maintains high density and dengue inhibition following introduction into a field population of *Aedes aegypti*. Philos Trans R Soc Lond B Biol Sci.

[21] Ryan PA, Turley AP, Wilson G, Hurst TP, Retzki K, Brown-Kenyon J (2019). Establishment of wMel *Wolbachia* in *Aedes aegypti* mosquitoes and reduction of local dengue transmission in Cairns and surrounding locations in northern Queensland, Australia. Gates Open Res.

[22] Lindsey ARI, Bhattacharya T, Newton ILG, Hardy RW (2018). Conflict in the intracellular lives of endosymbionts and viruses: a mechanistic look at *Wolbachia*-mediated pathogen-blocking. Viruses.

[23] Utarini A, Indriani C, Ahmad RA, Tantowijoyo W, Arguni E, Ansari MR (2021). Efficacy of *Wolbachia*-infected mosquito deployments for the control of dengue. N Engl J Med.

[24] Dos Santos GR, Durovni B, Saraceni V, Souza Riback TI, Pinto SB, Anders KL (2022). Estimating the effect of the wMe release programme on the incidence of dengue and chikungunya in Rio de Janeiro, Brazil: a spatiotemporal modelling study. Lancet Infect Dis.

[25] Carrington LB, Tran BCN, Le NTH, Luong TTH, Nguyen TT, Nguyen PT (2018). Field- and clinically derived estimates of *Wolbachia*-mediated blocking of dengue virus transmission potential in *Aedes aegypti* mosquitoes. Proc Natl Acad Sci USA.

[26] Nguyet MN, Duong TH, Trung VT, Nguyen TH, Tran CN, Long VT (2013). Host and viral features of human dengue cases shape the population of infected and infectious *Aedes aegypti* mosquitoes. Proc Natl Acad Sci USA.

[27] Fraser JE, De Bruyne JT, Iturbe-Ormaetxe I, Stepnell J, Burns RL, Flores HA (2017). Novel *Wolbachia*-transinfected *Aedes aegypti* mosquitoes possess diverse fitness and vector competence phenotypes. PLoS Pathog.

[28] Ferguson NM, Kien DT, Clapham H, Aguas R, Trung VT, Chau TN (2015). Modeling the impact on virus transmission of *Wolbachia*-mediated blocking of dengue virus infection of *Aedes aegypti*. Sci Transl Med.

[29] Frentiu FD, Zakir T, Walker T, Popovici J, Pyke AT, van den Hurk A (2014). Limited dengue virus replication in field-collected *Aedes aegypti* mosquitoes infected with *Wolbachia*. PLoS Negl Trop Dis.

[30] Hepworth G. Estimation of proportions by group testing. PhD dissertation. Melbourne: University of Melbourne; 1999.

[31] Walter SD, Hildreth SW, Beaty BJ (1980). Estimation of infection rates in populations of organisms using pools of variable size. Am J Epidemiol.

[32] Biggerstaff B. Mosquito surveillance software. Atlanta: Division Vector-Borne Diseases, US Centers for Disease Control and Prevention; 2007. https://www.cdc.gov/mosquitoes/mosquito-control/professionals/MosqSurvSoft.h. Accessed May 2022.

[33] Gu W, Lampman R, Novak RJ (2004). Assessment of arbovirus vector infection rates using variable size pooling. Med Vet Entomol.

[34] Hepworth G, Watson R. Debiased estimation of proportions in group testing. J R Stat Soc Ser C Appl Stat. 2009;58:105.

[35] Gu W, Lampman R, Novak RJ (2003). Problems in estimating mosquito infection rates using minimum infection rate. J Med Entomol.

[36] Hepworth G, Biggerstaff BJ (2021). Bias correction in estimating proportions by imperfect pooled testing. J Agric Biol Environ Stat.

[37] Biggerstaff BJ. Confidence intervals for the difference of two proportions estimated from pooled samples. J Agric Biol Environ Stat. 2008;13:478–96. 10.1198/108571108X379055.

[38] Garrett PE (2021). EP12-user protocol for evaluation of qualitative test performance.

[39] US Food and Drug Administration. Pooled sample testing and screening testing for COVID-19. 2020. https://www.fda.gov/medical-devices/coronavirus-covid-19-and-medical-devices/pooled-sample-testing-and-screening-testing-covid-19. Accessed Oct 2021.

[40] Pacidonio EC, Caragata EP, Alves DM, Marques JT, Moreira LA (2017). The impact of *Wolbachia* infection on the rate of vertical transmission of dengue virus in Brazilian *Aedes aegypti*. Parasit Vectors.

[41] Guo Y, Guo J, Li Y (2022). *Wolbachia* wPip blocks Zika virus transovarial transmission in *Aedes albopictus*. Microbiol Spectr.

[42] Hussain M, Zhang G, Leitner M, Hedges LM, Asgari S (2023). *Wolbachia* RNase HI contributes to virus blocking in the mosquito *Aedes aegypti*. iScience..

[43] Stouthamer R, Breeuwer JA, Hurst GD (1999). *Wolbachia pipientis*: microbial manipulator of arthropod reproduction. Annu Rev Microbiol.

[44] Mejia AJ, Dutra HLC, Jones MJ, Perera R, McGraw EA (2022). Cross-tissue and generation predictability of relative *Wolbachia* densities in the mosquito *Aedes aegypti*. Parasit Vectors.

