# Supplementary material for: Wolbachia wMel strain-mediated effects on dengue virus vertical transmission from Aedes aegypti to their offspring
Source: Parasit Vectors. 2023 Aug 31;16:308. doi: 10.1186/s13071-023-05921-y (PMC10472731; doi:10.1186/s13071-023-05921-y)
Supplement: Supplementary file 1 — Additional file 1: Table S1. Number of F1 adults distributed in each pooled sample. [file 13071_2023_5921_MOESM1_ESM.docx]

| Mosquito type | Pool size | Numbers of pooled samples | Numbers of DENV-1 pooled samples |
| --- | --- | --- | --- |
| Wt | 1 | 7 | 0 |
| Wt | 2 | 9 | 0 |
| Wt | 3 | 8 | 0 |
| Wt | 4 | 10 | 0 |
| Wt | 5 | 9 | 0 |
| Wt | 6 | 8 | 0 |
| Wt | 7 | 7 | 0 |
| Wt | 8 | 727 | 9 |
| *w*Mel | 1 | 16 | 0 |
| *w*Mel | 4 | 21 | 0 |
| *w*Mel | 8 | 675 | 0 |

Additional file 1: Table S1: Number of F1 adults distributed in each pooled sample
